# Letters to Physicians

**Published:** 1911-01

**Authors:** George M. Gould

**Affiliations:** Ithaca, N. Y.


### Letters to Physicians

### II. Epilepsy and "The Neuropathic Diathesis," "Heredity" and so forth

By GEORGE M. GOULD, M.D.
Ithaca, N. Y.

A WOMAN of 43 came to me about a year and a half ago giving a history of sick headaches, vomiting, and the like,—typical "migraine"—ever since early childhood. With this disease were associated in her case a constant miserable feeling in the head, "nervousness," considerable indigestion, capricious appetite, insomnia, pain in back of the neck, and other similar symptoms. Glasses by a "leading oculist" had been twice "fitted," but she "could not wear them." At her age and with this history I could promise little or no relief, at least for an indefinite time in the future and there was, for four months, but slight improvement. The first symptoms to disappear were as usual the vomiting and sickheadache. When the woman at her first visit described her symptoms she spoke of "fainting spells" (which I told her would disappear with correct spectacles), but she said nothing about epilepsy, nor anything except the swooning which might lead one to suspect that disease. How many patients avoid even telling their physicians of their epileptic seizures! How many cures unknown to the oculist or profession have been made by spectacles!

Sixteen months after the first visits she came in to consult me about her child, and something she said led me to quiz her closely about herself. I then secured an astonishing history of "typical" or old-fashioned epilepsy which had been concealed from me. It seems that her "fainting spells" had existed for twenty years or more occurring, usually, every day or two, sometimes not so frequently, and that they stopped four or five months after the first glasses had been ordered, and at the time those were replaced by others just a year ago. She now admitted that the "swoonings" were genuine epileptic seizures, with biting of the tongue, and the usual symptoms. There had been a slight switching of the axis of astigmatism in the four months after the first prescription; after this change every symptom of ill health disappeared entirely, and she has been perfectly well for the past year.

If the "change of life" increases ill health, as the popular superstition has it, then so much the worse for the superstition. If the symptoms were properly labeled "neurasthenia," then neurasthenia is due to eyestrain.

If it is suggested that typical epilepsy of twenty years chronicity is incurable, a big doubt is cast upon the ignorance, and blundering, and dogmatism of such therapeutic nihilism. If it is said that the eyestrain origin of much epilepsy is the foolish untrue theory of the oculistic hobby rider, one may answer—well, many things!

And if one says that it was the "neuropathic diathesis" and heredity,—that is, Fate,—which accounts for all such cases, then we aver that, barring the time of sick headaches and seizures, there was never any time when the woman was not apparently exceptionally rugged and of the healthiest appearance.

Of course, and again of course, her astigmatism, in the bitter untruth of the customary diagnostics, "was too little to correct."

My last prescription was:

R.+Sph. 0. 50 +Cyl. 0. 25 ax. 45° } Distance

L.+Sph. 0. 50 +Cyl. 0. 37 ax. 180° }

With plus spherical 1.00 added for man, in bifocals.

It was, indeed "too slight to correct" if not estimated correctly.

If it was not "too little to correct," it certainly argues the need of a revolution in the methods of diagnosticating it, and another resolution in the professional opinion as to the value of such slight causes of disease.

Perhaps the "Neuropathic Diathesis," or the Mumbo Jumbo of "Heredity" will account for the "fainting spells" of her little girl aged eight, whom this mother brought to me a year and a half ago. These faintings began at the age of 18 months; they lasted, each, about five or ten minutes, and usually occurred about every four days, sometimes less frequently. There was no biting of the tongue. They disappeared at once upon wearing the glasses I ordered, which were:

R.+Sph.1.25 +Cyl.1.00 ax.90° }

L.+Sph.1.37 +Cyl.0.87 ax.90° }

Her general health is now perfect; she had been small of stature but is now growing rapidly. She had decided lateral spinal curvature when she first came, but the swaying and postural exercises I ordered have made her spine straight and of perfect function.

